# A case report of a mammary myofibroblastoma in a male and literature review of radiologic and pathologic features of breast myofibroblastoma

**DOI:** 10.1002/ccr3.2413

**Published:** 2019-09-03

**Authors:** Majid Akrami, Saba Ebrahimian, Akbar Safaei, Zhale Tabrizi, Shadi Ebrahimian

**Affiliations:** ^1^ Breast Diseases Research Center Shiraz University of Medical Sciences Shiraz Iran; ^2^ Department of Surgery Lorestan university of Medical Sciences Lorestan Iran; ^3^ Department of Pathology Shiraz University of Medical Sciences Shiraz Iran; ^4^ Department of Radiology Isfahan University of Medical Sciences Isfahan Iran

**Keywords:** breast, myofibroblastoma, neoplasm, soft tissue

## Abstract

We reported a 65‐year‐old male with a benign mammary myofibroblastoma. This report shows that not all masses of male breast are malignant. To differentiate benign masses from malignant neoplasms, careful preoperative diagnosis should be performed. Preoperative diagnosis of the tumor prevents placing a huge financial and mental burdens on patients.

## 1 INTRODUCTION

Myofibroblastoma is a rare benign tumor and is observed in both males and females. The aim of this study was to examine this rare tumor and run a discussion about imaging, cytological, and histological features of the tumor. We presented a breast mass in a 65‐year‐old male, which was diagnosed as myofibroblastoma using immunohistochemical methods and managed surgically. The patient was followed for 5 years and no recurrence was observed.

Breast mass in males has many differential diagnoses including gynecomastia, infection, lipoma, granular cell tumor, metastatic disease, and schwannoma.^1^ Myofibroblastoma is another differential diagnosis of breast mass in males. It is a rare benign tumor derived from mesenchyme. Myofibroblastoma can occur anywhere in the body, but usually occurs in the breast. This tumor tends to be more prevalent in males than females.^2^ In this study, we described a case of breast myofibroblastoma in a male. We also reviewed the literature and highlighted the clinical presentations, as well as radiologic and pathologic features of mammary myofibroblastoma.

## 2 CASE HISTORY

A 65‐year‐old male presented to the office with a breast mass. He did not have family history of breast or ovarian cancer. He also did not use any drugs and was a nonsmoker. Physical examination revealed a round and nontender mass measuring approximately 4 cm in the upper part of the left breast. No nipple discharge or nipple retraction was observed in the physical examination. Moreover, there was no palpable lymphadenopathy and the right breast was normal. The patient underwent ultrasonography of bilateral breast and axillary area with 10 MHz probe. A 46*18 mm solid hypoechoic, well‐defined, and homogenous mass with small calcification at the upper part of the left breast was detected. No axillary lymphadenopathy was observed in ultrasonography.

The fine needle aspiration cytology (FNAC) of the breast mass was performed and it reported as follows. Smears showed abundant, randomly arranged single and clustered benign spindle‐shaped mesenchymal cells, with elongated or oval nuclei, displaying a finely granular chromatin pattern and inconspicuous nuclei. However, pleomorphism and mitotic activity were not observed and no epithelial component was identified.

**Figure 1:**
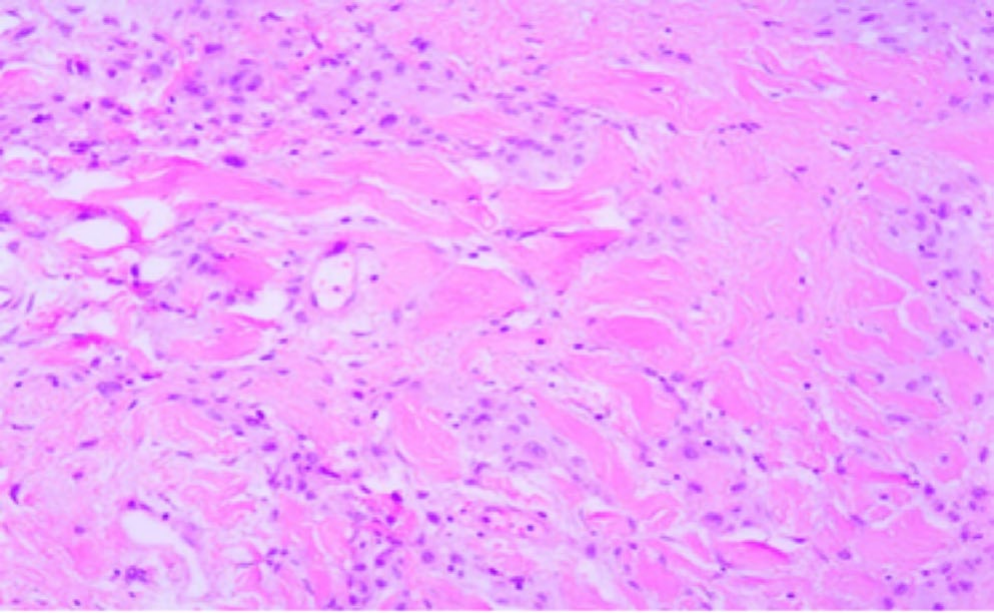
Nests of histiocytoid cells which are separated by collagen bundles H&E staining ×200)

**Figure 2:**
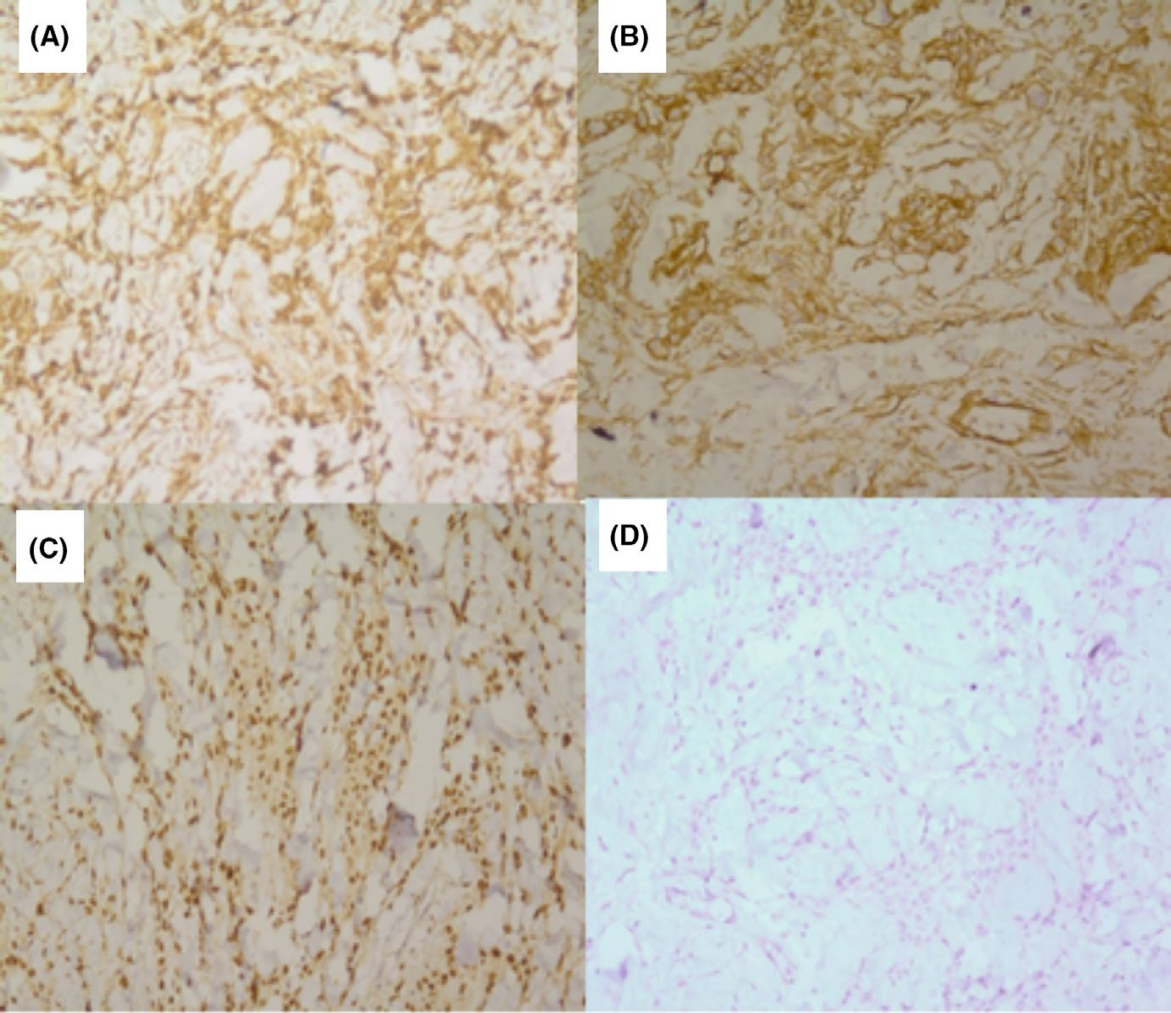
Immunohistochemistry staining. A, Positive for desmin. B, Positive for CD34. C, Positive for estrogen receptor. D, Negative for cytokeratin

The patient subsequently underwent modified radical mastectomy. The gross description of the mass was a well‐defined creamy‐white mass measuring 4*2.5*2.5 cm with a cavity next to it. The specimen consisted of the left breast product of modified radical mastectomy. Moreover, the cut sections showed a well‐defined, creamy‐white and firm mass measuring 4*2*2 cm. Three good samples were taken and sent to an expert pathologist for histopathological assessment. The histopathology of the surgical specimen showed nests of cells with spindle to oval nuclei and abundant amphiphilic cytoplasm without significant mitosis or pleomorphism which were separated by broad bands of hyalinized collagens (Figure 1). Further, immunohistochemistry staining revealed the positive reaction of epithelioid cells for desmin (D33), CD34 (QBEnd‐10), estrogen receptor (1D5) and progesterone receptor (PgR636) and negative reaction for CD31, cytokeratin and leukocyte common antigen (Figure 2). The final pathological diagnosis was a cellular variant of myofibroblastoma with no significant pathologic changes in margins, nipple, skin, and lymph nodes. The patient did not receive any adjuvant therapy and was followed every 6 months for 5 years. During the period, no recurrence was observed in the patient.

## 3 DISCUSSION

Myofibroblastoma is a rare benign breast tumor that consists of collagen bundles, adipocytes, and myofibroblastic stromal cells.^2^ It was first recognized in 1981 by Toker 3 and first described in 1987 by Wargotz et al, 4 who reviewed 16 cases of this tumor and named it myofibroblastoma. In that report, 11 patients out of a total 16 patients were men. Although some cases of mammary myofibroblastoma have been reported in women, most cases reviewed in the literature were in men between the ages of 41‐85 years.^3^, ^5^

Most cases of myofibroblastoma occur sporadically with no genetic predisposition. However, there are few case reports related to gynecomastia, chest wall trauma, surgical incision site scar tissue, and irradiation for breast cancer.^5^, ^6^, ^7^

The typical finding of myofibroblastoma is a slow‐growing solitary mass ranging from 1 to 4 cm. The clinical finding of the tumor is a solitary, unilateral, painless, mobile, firm, and nontender nodule.^8^

Ultrasonography is the first‐line diagnostic imaging, which is done in a patient with a breast mass. In ultrasonography, myofibroblastoma is a well‐circumscribed, solid, round to oval, hypoechoic heterogeneous mass with variable posterior attenuation. Although internal vascularity may be observed in Doppler, architectural distortion and calcium are not commonly seen in ultrasonography.^9^, ^10^ However, in our case, calcification was detected in ultrasonography.

The ultrasonographic findings of myofibroblastoma are similar to those of fibroadenoma in women and angiolipoma, fat necrosis, and pseudoangiomatous stromal hyperplasia in men, considering myofibroblastoma as a differential diagnosis of a mass with benign sonographic features.^11^, ^12^ Moreover, the mammographic characteristics of myofibroblastoma are nonspecific findings, including a heterogeneous mass with a well‐defined border and no evidences of microcalcification.^13^

Although magnetic resonance imaging (MRI) is not routinely used in diagnosis of a breast mass, there are some reports of the features of myofibrobalastoma in MRI. The review of the MRI findings revealed the myofibroblastoma as a tumor with a hyperintense signal in T2‐weighted images surrounded by a hypointense capsule or nonenhancing internal septation in postcontrast imaging.^12^, ^14^, ^15^ In addition to the mentioned MRI characteristics, ADC has been reported as a useful MRI finding in distinguishing myofibroblastoma form malignant masses. Since low values of ADC are detected in malignant lesions, high ADC values in myofibroblastoma may be helpful in differentiation of myofibroblastoma from malignant lesions.^16^ However, Yilmaz et al did not find the ADC value as a helpful index in differentiating myofibroblastoma from breast cancer.^12^

In addition to the mentioned diagnostic imaging methods, breast myofibroblastoma has been reported as an incidental finding in computed tomography (CT). It was described as a low or mixed low and high density mass on CT.^17^, ^18^, ^19^ Although CT is not used as a diagnostic workup of mammary myofibroblastoma, it can be performed to exclude involvement of proximal structures.

Since the imaging features of myofibroblastoma are nonspecific, the gold standard method for diagnosis of myofibroblastoma is histopathology.

The FNAC findings of myofibroblastoma have been rarely reported.^20^, ^21^, ^22^ Myofibroblastoma of the breast can be suspected preoperatively using FNAC. The cytological clues include oval to spindle‐shaped cells arranged randomly in a variable myxoid collagen matrix, extended slightly moderately cellular, fascicular clusters, naked ovoid nuclei, scant cytological atypia, nuclear groove, and intranuclear pseudoinclusions, absence of epithelial elements and absence of mitosis and necrosis.^21^, ^23^, ^24^ Our case demonstrated the same features in FNAC including spindle cells with arranged single and clustered benign spindle‐shaped mesenchymal cells with a finely granular chromatin pattern and with no signs of pleomorphism, mitotic activity, and epithelial component.

Most cases of mammary myofibroblastoma can be diagnosed using a conjunction of clinical, radiologic, and FNAC features. However, in some cases, diagnosis remains ambiguous and needs more diagnostic workups including core biopsy or excisional biopsy.^7^

In the macroscopic view, the cut of a myofibroblastoma shows a well demarcated pink or tan round mass.^25^ In histopathology, myofibroblastoma is presented as some bipolar spindle‐shaped cells showing variable myogenic and fibroblastic differentiation in short intersecting fascicles interrupted by keloidal like eosinophilic collagen bands.^25^, ^26^ In immunohistochemistry, myofibroblastoma stains positively for Vimentin, Desmin, and CD34. Positively stained with estrogen, progesterone, androgen receptors, SMA, S100, bcl‐2 and negatively for CD31, and cytokeratin are detected in some types of myofibroblastoma.^24^

Myofibroblastoma can show different variants using histological patterns. Variants identified included classic, cellular, collagenous/fibrous, lipomatous, infiltrative, myxoid, epithelioid, and decidua‐like variants.^26^ Epithelioid type and cellular type of myofibroblastoma can mimic the histologic features of invasive lobular carcinoma and metaplastic breast carcinoma, respectively. The negative staining for cytokeratin can help in differentiating these tumors 27, 28

The long term prognosis is good for myofibroblastoma, and excisional biopsy is usually sufficient for the treatment of myofibroblastoma.^9^ No metastasis or malignant transformation of myofibroblastoma has been reported in the literature. Recurrence has only been reported in a 25‐year‐old female with bilateral myofibroblastoma.^5^

## CONFLICT OF INTEREST

None declared.

## AUTHOR CONTRIBUTION

SHE and SE and MA and ZT gathered the patient's data and collected materials. SHE prepared the review and wrote the manuscript. All authors read the final manuscript.
